# Obstetrical Problems

**Published:** 1884-08-20

**Authors:** 


### OBSTETRICAL PROBLEMS.

It is singular what mistakes physicians fall into from a want of ability to co-ordinate their thoughts. I have no doubt but many of my readers—shall I say *most* of them?—have been picked up in some ridiculous way at some time or other. I do not claim to be any exception to the rule, and in early days I had my experiences.

A very singular one came to me in my second year. The woman had been delivered without unusual suffering, and everything seemed well, and the doctor left in an hour feeling very comfortable. In some eight hours there was an urgent call; the woman was suffering intense pain—it was real agony. I gave macrotys, diaphoretic powder, gelseminum, and at last morphine, but without the slightest relief. I had exhausted myself, and was in a pocket. The woman was suffering so severely that something must be done, and, as I could not do better, I called my regular neighbor—a very clever fellow, by-the-by. I described my case, and he said, "Doctor, I suspect that there is a small piece of placenta, a shred of the membranes or a dense clot engaged in the os, and she will not be relieved until it is removed." And so it proved. A piece of placenta half as large as my little finger had been torn off, and all this suffering was due to this, and its removal gave prompt and permanent relief. This was one of the best lessons I ever learned, and many is the time that it has been put in practice.

I was called two hundred and fifty miles to see a patient who had suffered intensely, and was now growing an unpleasant metritis, from the retention of a portion of the membranes. They were removed without much difficulty, and with the membranes came three or four ounces of putrid blood. The patient had marked relief at once, and, with small doses of chlorate of potassium, made a good recovery.

I have seen and been consulted in so many such cases that I have been forced to believe them quite common. Now it is a small

*A Southern "gin-house" is the building where the cotton is *ginned* or cleared of seeds and packed into bales for market, and a peculiar odor hangs around it, known only to Southerners or those who have seen one.

piece of placenta, then a shred of the membranes or a clot of blood. If the severe pains are sufficient for the expulsion, well; if not, there is uterine irritation, unpleasantness, puerperal fever, or a protracted semi-purulent and offensive discharge, with sub-involution and great impairment of the general health.

Make it a rule to carefully remove the secundines, and see that the uterus is firmly contracted. Afterwards see that blood clots and lochial discharge do not accumulate, or are removed if there is increased suffering or fever. I am not sure but what the advice of a celebrated New York physician is good: "Let the woman attend to the calls of nature in the natural position, and uterus and vagina will free themselves from much offeuding material."

This will answer one of our readers who says: "I had a very unpleasant obstetric case lately. The labor was all right, but the woman suffered the worst after-pains I ever saw, and the discharge became so offensive that persons could hardly stay in the room. On the eighth day she passed something like the skin of an egg, and then commenced to improve. What was it?"

It is not an uncommon thing for the os to be lost, or, at least, the doctor with the "wabbling" mind and uncertain finger fails to find it. In the majority of cases this rectifies itself as the labor advances, and the uncertain doctor at last finds an opening when he was sure it had grown up. Where is the os? Looking backward into the hollow of the sacrum, waiting for a long finger to bring it forward. Here is a case in point:

"A question for the 'Journal' came up here a short time ago. A young woman of twenty, tall, strong-looking, expected a baby—at term. She got great pain in her head, became blind, and convulsions came on. Two doctors called. Os could not be found for twenty-four hours; patient was bled, emeticked, injected, etc.; finally the os was found and, after two days fuss, dilated in some way; child 'bored' out, and woman will recover."

What should be the practice? It is as plain as the nose on the doctor's face. Go back into the hollow of the sacrum and find the os. If the finger is too short, press the perineal tissues up, take advantage of the genital fissure, and reach it. If this does not answer, take two fingers, or the whole hand if necessary. Hook the finger in the os, and, with the hand on the fundus to elevate it, draw the os forward into the axis of the pelvic cavity. Now the labor progresses well.

I have had many such cases in my practice, and have seen a half dozen or more cases in consultation. I recall one case in which the woman had been in labor thirty-six hours. The doctor was confounded, and had so lost himself that he could not tell which end of the patient was at fault. The pain had nearly stopped. It took considerable finger to reach the offending opening, but it was brought to the center of the pelvis, when, with one continuous pain, lasting some fifteen minutes, the head was expelled.

I should not be willing to say that the physician in attendance could have had a like result twenty-four hours before, for the best obstetricians may be mistaken as to time, but I think it possible.

One of the funniest cases of occluded os I ever saw was one of tough membranes. The attendant could find no opening, look where he would, and his finger passed over a smooth, unyielding surface, which he was sure was the lower segment of the uterus. He had been holding it back, telling the woman not to bear down until assistance could get to the house. This, of course, was a good case, and was brought to a speedy termination.

One is inclined to laugh at such cases, untill he recalls some of his own experiences, when *he* was in doubt, and where Nature solved the doubt and the difficulty.

I was called on some two months ago to help a couple of young physicians out of trouble. The labor had been natural, and the child was delivered at four o'clock in the morning. It was now after nine and the placenta was not delivered. They had done what they could to this end, and there had been considerable hemorrhage. They thought it had "grown fast."

It was the simplest of troubles. The placenta had been detached in the usual way, the os was well contracted, and it lay over the opening like a button in its button-hole. No amount of traction on the cord or bearing down of the woman would remove it. But it could be readily *unbuttoned* by passing a finger over the anterior edge and bringing it down.

One of the hardest journeys I ever made was on an occasion like this. Sixty miles by rail and twenty by wagon, over the roughest of countries. The doctor had pulled the cord off in his vain efforts, and it required the introduction of the greater part of the hand to get a fair hold upon the placenta and bring its edge down and deliver it.

I will name but one thing more, and it should never need naming to educated physicians. This is the necessity of cleanliness of the genital passages and outlet. Many puerperal women are left in their filth. Washing is thought to be very injurious, as is sitting up to urinate. The vulva and its adornments, with nates and thighs, become soiled with the discharges, and many times with clotted blood, and the nastiness is sometimes so great as to breed local inflammation and, by absorption, an unpleasant feverishness and impairment of the blood.

Whilst I cannot agree with those asinine writers who wish to syringe the uterus in every case, and every day after confinement, it is well to insist that these parts be washed with warm water and soap, and if there is the slightest odor or irritation of the soft parts, a solution of borax is an admirable thing.

Would you believe that maggots could be bred in the cloths applied to the vulva, and in that used for the protection of the bed? No! Well, I have seen such things, and even within the genital fissure. We do not want any of this in our practice, and we learn to detest dirt as we shun the devil.—*Eclectic Med. Journal.*

---

Philadelphia News: Oscar Wilde says we should beautify our kitchens. That is all very nice in theory, but if a man hires a very pretty cook there is apt to be trouble in the family. Better let the kitchen alone.

